# Sleep defined by arousal threshold reveals decreases in corticocortical functional correlations independently from the conventional sleep stages

**DOI:** 10.1101/2024.08.09.607376

**Published:** 2024-08-13

**Authors:** Dante Picchioni, Fan Nils Yang, Jacco A. de Zwart, Yicun Wang, Hendrik Mandelkow, Pinar S. Özbay, Gang Chen, Paul A. Taylor, Niki Lam, Miranda G. Chappel-Farley, Catie Chang, Jiaen Liu, Peter van Gelderen, Jeff H. Duyn

**Affiliations:** 1 Advanced Magnetic Resonance Imaging Section, National Institute of Neurological Disorders and Stroke, USA; 2 Department of Radiology, Stony Brook University, USA; 3 Artificial Intelligence for Image-Guided Therapy, Koninklijke Philips NV, Netherlands; 4 Institute of Biomedical Engineering, Boğaziçi University, Turkey; 5 Scientific and Statistical Computing Core, National Institute of Mental Health, USA; 6 School of Medicine and Dentistry, University of Rochester, USA; 7 Center for Sleep and Circadian Science, University of Pittsburgh, USA; 8 Departments of Electrical Engineering and Computer Science, Vanderbilt University, USA; 9 Advanced Imaging Research Center, University of Texas Southwestern Medical Center, USA

**Keywords:** functional magnetic resonance imaging, arousal thresholds, sleep stages, linear mixed effects

## Abstract

Sleep research and sleep medicine have benefited from the use of polysomnography but have also suffered from an overreliance on the conventional, polysomnography-defined sleep stages. For example, reports of sleep-specific brain activity patterns have, with few exceptions, been constrained by assessing brain function as it relates to the conventional sleep stages. This limits the variety of sleep states and underlying activity patterns that one can discover. If undiscovered brain activity patterns exist during sleep, then removing the constraint of a stage-specific analysis may uncover them. The current study used all-night functional magnetic resonance imaging sleep data and defined sleep behaviorally with auditory arousal threshold (AAT) to begin to search for new brain states. It was hypothesized that, during sleep compared to wakefulness, corticocortical functional correlations would decrease. Functional correlation values calculated in a window immediately before the determination of AAT were entered into a linear mixed effects model, allowing multiple arousals across the night per subject into the analysis. The hypothesis was supported using both correlation matrices of brain networks and single seed-region analyses showing whole-brain maps. This represents a novel approach to studying the neuroanatomical correlates of sleep with high spatial resolution by defining sleep in a way that was independent from the conventional sleep stages. This work provides initial evidence to justify searching for sleep stages that are more neuroanatomically localized and unrelated to the conventional sleep stages.

## Introduction

To understand the brain, scientists and clinicians must use a variety of complementary neuroscientific measurements that are optimized for different temporal and spatial resolutions (Huettel et al., 2014). This is particularly important for sleep research and sleep medicine, which has benefited from the advent of electro-encephalography (EEG) but has also suffered from an overreliance on the conventional EEG sleep stages (Decat et al., 2022; Duyn, 2012; Himanen & Hasan, 2000; Hori et al., 1994; Kay et al., 2017; Moehlman et al., 2019; Nir & Tononi, 2010; Nofzinger, 2004; Prerau et al., 2014). EEG provides excellent temporal resolution, whereas functional magnetic resonance imaging (MRI) provides good spatial resolution and can localize brain activity with much better spatial fidelity than scalp EEG. Neuroanatomical localization is important because it enables the detection of local sleep (e.g., Jang et al., 2022; Krueger & Obal, 1993) and facilitates the linkage of the various brain states that occur during sleep and the neural mechanisms underlying these states to the functions of sleep (Maquet et al., 1997). Functional MRI can thus provide a parallel avenue of research to complement EEG sleep research. Sleep stages are defined with polysomnography (PSG), but the term EEG will be used for simplification, to contrast it better with other techniques, and because it is the most important measure in PSG.

The conventional sleep stages are defined with EEG, but sleep itself is not. Sleep is defined by four widely accepted behavioral characteristics: species specific posture, absence of motion, increased arousal threshold, and immediate reversibility (Flanigan et al., 1973; Kleitman, 1963; Piéron, 1913). To determine arousal threshold in humans, researchers typically deliver auditory stimuli of increasing intensity during sleep, and sleep depth is quantified with the intensity that was necessary to produce an unambiguous behavioral response. The EEG became the gold standard for defining sleep because when it was invented, researchers discovered a strong correlation between scalp EEG 0.5–3.0 Hz waves and auditory arousal threshold (AAT; Blake & Gerard, 1937). Thus, the EEG is a *surrogate* for the behavioral definition of sleep rather than a true gold standard. The continued importance of the behavioral definitions is best demonstrated when investigators must define sleep in novel model organisms or at early developmental stages (e.g., Blumberg et al., 2005; Hendricks et al., 2000) or when attempting to combine techniques synergistically (e.g., Prerau et al., 2014). One might guess that when other techniques were invented to measure the brain during sleep, the first experiments would have been to perform correlations with arousal threshold. These experiments have never been performed, either with functional MRI or with any other modern technique.

Many have measured brain function during sleep with modern techniques such as positron emission tomography (Maquet, 2000), functional MRI (Picchioni et al., 2013), and other techniques (Tarokh et al., in press). However, with few exceptions, such as when investigators examined phasic EEG events (e.g., Fogel et al., 2017) or delivered stimuli to study evoked neural responses (e.g., Portas et al., 2000), all of these studies reported sleep-specific brain activity/functional correlation patterns constrained by the conventional sleep stages. This limits the variety of sleep states and underlying activity patterns that one can discover. As an aside, functional correlations are colloquially called functional connections, but throughout this manuscript the former term will be used because it is more precise.

Based on this gap in the literature, if undiscovered spatiotemporal patterns of activity/functional correlations exist during sleep, one would make the following prediction: when studying one conventional EEG sleep stage, investigators should observe different brain and behavioral results within this stage or between a conventional stage and a new stage that covers the transition between the two. This has been found with arousal thresholds between tonic and phasic stage rapid eye movement (REM) sleep (Ermis et al., 2010; Price & Kremen, 1980), with unique theta fluctuations across stage REM sleep in mice and humans accompanied by uniquely patterned cortical waves in mice (Bueno-Junior et al., 2023; Dong et al., 2022), with changes in autonomic nervous system tone within stage nonrapid eye movement (NREM) 2 sleep (Brandenberger et al., 2005), with differing neuroimaging brain activity/functional correlation patterns within the same conventionally defined sleep stage (Guo et al., 2023; Picchioni et al., 2008; Stevner et al., 2019; Watanabe et al., 2014; Wehrle et al., 2007; Yang et al., 2024), and with a NREM sleep stage characterized by ponto-geniculo-occipital waves occurring immediately before the transition to stage REM sleep in rats (Datta, 2000). These data indicate that undiscovered patterns of activity/functional correlations indeed exist during sleep, motivating an expanded perspective on the definition of a sleep stage. A conventional sleep stage is defined as a pattern of scalp electrical activity associated with higher arousal thresholds and different amounts of dreaming. If this definition is expanded to include any neuroanatomically specific pattern of brain activity/functional correlations available from advanced methods throughout the full spatial extent of the brain, it would be unsurprising to find that new sleep stages have always existed and remained undiscovered only because the standard measurement techniques in the field were limited. The current study used novel all-night functional MRI sleep data (Moehlman et al., 2019) and defined sleep behaviorally with AAT to begin to search for those patterns. The goal was not necessarily to discover those patterns but to provide initial evidence to justify searching for sleep stages that are more neuroanatomically localized and unrelated to the conventional EEG sleep stages.

It was hypothesized that, during sleep (defined by high AATs) compared to wakefulness (defined by low AATs), corticocortical functional correlations would decrease. This finding would replicate prior research (Picchioni et al., 2013) and fill a critical gap by establishing the utility of functional MRI as a tool for searching for new sleep stages that can include any neuroanatomically specific pattern of brain activity/functional correlations.

## Methods

### Overview

Data were acquired during a pilot study. This study was designed to test hardware and software, establish procedures, and estimate effect sizes for an *a priori* statistical power analysis for a subsequent main study. Substantive results for the pilot study are reported here given the long duration and complexity of the overall study and to perform data-driven analyses that can be applied to the data from the main study. These results have been posted to a preprint archive (Picchioni et al., 2024). A description of the general method, its feasibility, and its validation has been published, along with a detailed description of the subjects, screening procedures, home-monitoring period, adaptation night, functional MRI data collection, simultaneous EEG data collection, and sleep staging (Moehlman et al., 2019).

An active noise cancellation system was used (OptoActive, OptoAcoustics, Mazor, Israel) to attenuate the scanner acoustic noise and deliver the auditory tones. Tones were delivered approximately eight times throughout the night to collect AAT as a behavioral measure of sleep depth. Tones were generated and delivered with Presentation (NeuroBehavioral Systems, Berkeley, USA). Tones were 1.25 kHz, which was chosen to be similar to the 1.0 kHz frequency typically used in human AAT studies (e.g., Bonnet et al., 1978; Chervin et al., 2009; Wills & Trinder, 1978) while avoiding harmonics of the 0.5-kHz functional MRI acoustic noise fundamental frequency. Each tone consisted of a sequence of five 0.1-s stimuli, separated by 0.5-s intervals.

The method of limits was used. A tone was delivered and, if the subject did not arouse, another tone was delivered 21 s later with a 5-dB increase. This continued until subjects gave a specific, arranged verbal response: “I’m awake.” If subjects did not arouse to 120 dB, a threshold of 125 dB was assigned as the finding, an approach that mirrors prior work (Wills & Trinder, 1978, p. 59; Zepelin et al., 1984, p. 296).

The intensity of the first tone was customized for each subject and each night. This was determined during wakefulness before lights-off by administering a hearing test with subjects in the MRI while replicating the same experimental procedures that would occur during sleep, including the use of insert ear plugs and functional MRI data collection. This test began with a subthreshold tone. The intensity was increased until subjects reported hearing the tone. This was repeated with a suprathreshold tone. Its intensity was decreased until subjects no longer reported hearing the tone. Rounding down to a 5-dB level, the average of the two was used as the waking perceptual threshold. AAT was defined as dB above the waking perceptual threshold.

Investigators from prior human AAT studies have used 2–16 arousals per night (Blake & Gerard, 1937; Bonnet et al., 1978; Bonnet & Moore, 1982; Bonnet et al., 1979; Busby & Pivik, 1985; Busby et al., 1994; Chervin et al., 2009; Goodenough et al., 1965; Johnson et al., 1979; Keefe et al., 1971; Kohlschutter, 1862; Michelson, 1897; Rechtschaffen et al., 1966; Rosenthal et al., 1996; Watson & Rechtschaffen, 1969; Wills & Trinder, 1978; Zepelin et al., 1984; Zimmerman, 1970). The mean value from these studies was approximately 6.4 arousals. Some variation in the number was expected when, for example, scheduled arousals were missed when troubleshooting equipment or accommodating requests for breaks from subjects. Therefore, arousals were scheduled to occur eight times per night so that the final number would approximate the average from prior studies.

Half of the arousals were scheduled to occur randomly throughout the night. For the other half, for these pilot data, the schedule was slightly biased towards stage NREM 3 sleep to compensate for the relative paucity of very high AATs under the current experimental conditions. Eight random numbers between 5 and 475 with replacement were generated at the beginning of the night. These represented minutes from lights off and corresponded to the planned arousals. When two numbers were within 10 minutes of each other, the second was rerandomized to prevent frequency bunching. When subjects entered stage NREM 3 sleep, the upcoming planned arousal was diverted to a stage NREM 3 sleep arousal using a second set of random numbers. This was possible because EEG data were collected to establish the method and could be manually sleep scored in real-time because real-time MRI artifact corrected EEG data were available (Moehlman et al., 2019). The second set of random numbers ranged from 1 to 10 min with replacement and represented the interval between the observed epoch of stage NREM 3 sleep and the new time for the next arousal. This allowed the arousals to be biased towards stage NREM 3 sleep while still retaining some randomness. Upon completion of this diverted arousal, one of the arousals planned at the beginning of the night was removed from the queue. This was done in reverse order from which the initial random numbers were generated. This continued until four arousals planned at the beginning of the night were replaced.

Functional MRI data were collected as previously described (Moehlman et al., 2019). Recording of peripheral signals was synchronized with functional MRI data collection with a volume trigger provided by the scanner. Concurrently acquired peripheral signals included a chest respiratory effort belt (to calculate respiratory flow rate) and a finger skin photoplethysmograph (to calculate peripheral vascular volume). They were acquired using AcqKnowledge with TSD200-MRI and TSD221-MRI transducers and an MP150 digitizer sampling at 1.0 kHz (Biopac, Goleta, USA).

Sleep scans varied in length between 5 min and approximately 3 hr, and they were terminated by either a break request from the subject or an experimental arousal with an AAT determination. The 4 min of data before the first tone of each arousal was used for all analyses. Windows as small as 30 s can be used in studies of dynamic functional correlations during sleep (Wilson et al., 2015). Using the data immediately before the first tone ensured the functional correlations would be tightly associated with the functional state that led to the AAT without being influenced by ascending state transitions or evoked activity. Only runs from the second night with ample, good-quality functional MRI and peripheral data were analyzed. This resulted in 77 four-min segments across 12 subjects.

Using a nearly identical approach compared to prior work on cerebrospinal fluid pulsations (Picchioni, Ozbay, et al., 2022), the functional MRI data were processed using AFNI Version 21.1.07 or later (Cox, 1996) with its afni_proc.py program (Reynolds et al., 2024; Taylor et al., 2018) in the following order. Outliers (spikes), polynomial trends, and two harmonics of the cardiac and respiratory signals from a modified “retrospective correction of physiological motion effects” model (Glover et al., 2000) were removed (see description of estimation, below). Slice-timing correction, motion registration, and nonlinear alignment (both nonlinear alignment to template space and anatomical skullstripping were performed with AFNI’s @SSwarper prior to running afni_proc.py; Cox & Glen, 2013; Saad et al., 2009) to the Talairach template (Talairach & Tournoux, 1988) were performed. For functional-structural alignment, a local Pearson correlation cost function, which is optimized for aligning datasets of differing tissue contrasts (Saad et al., 2009), was applied, as well as local “unifizing” of the functional data to reduce effects of any brightness inhomogeneity. For functional data motion correction and structural data alignment, the functional volume with minimum outlier fraction (“MIN_OUTLIERS”) was selected as a reference (Reynolds et al., 2024). The estimated motion parameters and their derivatives were removed using 0/1 weighted regression to ignore (censor) any time points with excessive motion, defined as follows. An aggregate measure of head motion was constructed by adding the *SD* of the 6 motion parameters (3 translations in mm and 3 rotations in deg). Volumes with this aggregate (summed) *SD* exceeding 0.3 units were censored. All subsequent analyses used the same weighted-regression approach. The final voxel size from this processing was 2.0 mm isotropic. The afni_proc.py command for one run for one subject is publicly available at https://github.com/dantepicchioni/sleep_pilot_afni_proc.py.

The “retrospective correction of physiological motion effects” modelling aims to remove fast, pulsatile artifacts in the functional MRI data; it does not remove artifacts caused by slower changes in autonomic nervous system tone. These were removed by calculating and separately regressing out two additional peripheral measures (Duyn et al., 2020). These variables were calculated from the respiratory effort and finger photoplethysmography signals as follows. To remove artifactual spikes from the data, time points exceeding four *SD* above the mean were set to the mean. Low-pass filtering was performed on the respiratory effort and photoplethysmography signals at 30 and 10 Hz, respectively. From the photoplethysmography signal, an indicator of peripheral vascular volume was created by calculating the *SD* of the signal in 3-s segments (the MRI volume repetition time); this is a measure of the excursion amplitude caused by the heart beat and is proportional to the volume of blood detected by the sensor (Allen, 2007). The filtered respiratory signal was used to derive a measure of respiratory flow rate, which previously was found to affect the functional MRI signal throughout the brain (Birn et al., 2008), albeit not significantly (Tu & Zhang, 2022). This was done by taking the derivative of the low-pass filtered signal, rectifying it, and applying a second low-pass filter at 0.13 Hz. To regress out the respiratory flow rate and peripheral vascular volume signals, two time-shifted versions for each. Shifts of 12 and 15 s were used for respiratory flow rate and 0 and 3 s were used for peripheral vascular volume. These values were expected to be effective in accounting for peripheral contributions to the functional MRI signal (Ozbay et al., 2019). To reduce the effect of outliers, the regression was performed in five-minute segments by zero-padding the regressors at five-minute intervals.

Simultaneous EEG data were collected and sleep staged as previously described (Moehlman et al., 2019). The modal sleep stage in the four minutes prior to the first tone was assigned as the sleep stage for that arousal. If one stage did not obtain a majority, the sleep stage immediately before the first tone was used.

### Experiment 1

Functional MRI can simultaneously measure almost the entire brain. To capitalize on this strength and to search for novel patterns during sleep, correlation matrices were created where every pairwise combination of network correlation was presented. In this context, a network is defined as a group of regions. This allowed 1) easy digestion of multiple relationships, 2) determination of how these relationships change with AAT, and 3) detection of the effect of any residual artifacts. These matrices are important and illustrative but are limited because it is challenging to gain an appreciation of correlations with and in specific brain regions. In other words, its spatial resolution is the primary advantage of functional MRI over scalp EEG, so it is important to capitalize on it by showing whole-brain maps alongside correlation matrices of average network activity. Three exemplar regions were chosen to create seed-region correlation maps of the relationship with AAT.

For the network analysis, a custom Talairach-space atlas was created by beginning with the Eickhoff-Zilles atlas (Eickhoff et al., 2005). The original atlas had 116 cortical and subcortical regions, which were created using both cytoarchitectural data and multiple task-based functional-activation maps. The cerebellar vermis regions were divided into left and right regions to facilitate the later creation of superordinate network categorizations because, although analogous to the corpus callosum in the neocortex, the vermis has a higher density of gray matter. The total was 124 left and right regions of interest.

These regions were manually categorized into 26 left- and right-hemisphere networks. In contrast to creating networks based solely on resting-state intrinsic correlation networks, this approach facilitated the inclusion of subcortical networks, allowed the potential analysis of both networks and regions, and had fewer assumptions because the regions were created from anatomy and activations associated with specific, ecologically relevant tasks. The manual categorizations were based on well-established networks (Menon, 2011; Raichle, 2011; Yeo et al., 2011) and reasoned categorizations by others (Andrews-Hanna et al., 2010; Mesulam, 1998; Vernet et al., 2014). For example, primary sensory and motor cortices were classed into a unimodal cortical network. The resulting networks are labelled as (1) default-mode network posterior cingulate cortex-anterior medial prefrontal cortex core, (2) default-mode network dorsal medial prefrontal cortex subsystem, (3) default-mode network medial temporal lobe subsystem, (4) dorsal attention, (5) ventral attention, (6) executive control, (7) language, (8) salience, (9) heteromodal misc, (10) unimodal cortical, (11) thalamus, (12) basal ganglia, and (13) cerebellum. Although it is allocortex, as others have done, the hippocampus was categorized with neocortical regions in the default-mode network medial temporal lobe subsystem because its activity has a strong positive correlation with other regions in this subsystem (Andrews-Hanna et al., 2010). Although the amygdala is typically not included in this subsystem, it was nevertheless added given its tight association with the functions of this subsystem. This atlas is publicly available at https://osf.io/uqrab/?view_only=2ae81bc515434bcc91901dab12c2b423.

The spatial mean of time points for the voxels in a network was calculated at each time point. This resulted in one functional MRI time series per network per run. No spatial smoothing was used. Spatial smoothing can be considered moot in this analysis because voxels are averaged across a large spatial extent for each network. This took the place of spatial smoothing, provided similar statistical advantages, and provided a similar control for the residual geometric distortion that is present in all functional MRI data.

All pairwise correlations between network time series were calculated with AFNI’s 3dNetCorr (Taylor & Saad, 2013). The program was used to output the Fisher *z*-transformed correlation values because the original Pearson *r* sampling distribution is skewed and bounded. The transformation normalizes it but results in values beyond *±* 1.0. The absolute value for each transformed correlation was computed because a strong positive correlation and a strong negative correlation would both indicate the same magnitude of assumed neural communication/information exchange between two brain networks (e.g., Picchioni et al., 2014; Zalesky et al., 2010). This approach is particularly appropriate when considering that activity in all regions of the brain can be evoked with a task, including default-mode network regions (e.g., Gusnard et al., 2001; Spreng et al., 2009).

Separately for each correlation, the transformed absolute correlation values were entered into a linear mixed effects analysis whereby each subject can contribute more than one pair of observations (i.e., a cluster of observations for each subject). The variabilities in the slopes and intercepts across the clusters are modeled with linear mixed effects by including both the individual and cluster intercepts and slopes in the regression. The fixed effects were one discrete, within-subject variable (condition with approximately six levels representing arousals across the night such as Arousal 1, Arousal 2, etc.) and one continuous, within-subject variable (AAT). The random effects were AAT and the intercept. The criterion variable was the correlation between any two networks.

The initial linear mixed effects analysis was performed with the fitlme function in MATLAB R2022a or later (The Mathworks, Natick, USA). The version with multiple testing correction was performed with R (Version 4.2.2 or later; R Core Team, 2022) and its Network-Based R-Statistics package (Research Resource Identifier: SCR_019114; Gracia-Tabuenca & Alcauter, 2020). The multiple testing correction is based on the network-based statistics approach (Zalesky et al., 2010), which is analogous to the voxel cluster approach used in brain maps.

The effect size used was Cohen’s *f* ^2^. Its estimate was calculated by subtracting the proportion of variance explained by *R*^2^ in a reduced model without AAT from a full model with AAT. *R*^2^ was ordinary and conditional.

For the seed-region analysis, bilateral hippocampus, thalamus, and posterior cingulate cortex were chosen as exemplar regions. The hippocampus was chosen given its relevance to sleep-dependent memory consolidation (e.g., Rasch et al., 2007), the thalamus was chosen given its relevance to sleep regulation (e.g., Steriade & McCarley, 2005), and the posterior cingulate cortex was chosen given its putative role in dreaming (e.g., Picchioni, Abdollahi, et al., 2022).

The spatial mean for the voxels in a seed region was calculated at each time point. The data were then spatially smoothed to 4 mm full width at half maximum. The vectors representing each seed region’s spatial mean across time were then correlated with all voxels in the brain. Identical to the network analysis, absolute values of the resulting Fisher *z*-transformed correlations were fed into a linear mixed effects analysis with AAT. In contrast to the network analysis, AFNI’s 3dLMEr (Chen et al., 2013) was used to enter whole-brain maps as the inputs into the linear mixed effects model.

Multiple testing correction was performed on the group-level maps as follows. After the correlations with the seed regions were calculated, the residuals were computed and the total extent of spatial smoothness in the residuals for each subject was calculated using non-Gaussian filtering (Cox et al., 2017a, 2017b). This obviates the minor problem associated with assuming normality in the smoothness of neuroimaging data (Eklund et al., 2016). These smoothness estimates in the residuals were averaged across all subjects and fed into AFNI’s 3dClustSim program, which uses Monte Carlo simulations to estimate the size of false-positive clusters. The following parameters were used: a two-sided test, an individual-voxel *α* = 0.05, a cluster-size *α* = 0.05, and a clustering technique of nearest neighbor with 26 face, edge, and cornerwise neighbors. This gave a minimum cluster size of 1,226, 1,195, and 1,203 voxels for the hippocampus, thalamus, and posterior cingulate cortex, respectively. Calculation of Cohen’s *f* ^*2*^ was identical to the network analysis. The only difference was r.squaredGLMM from the MuMIn R package was used to calculate *R*^2^ within AFNI’s 3dLMEr program. Unthresholded statistical brain maps were masked to exclude voxels in white matter and cerebrospinal fluid.

### Experiment 2

In this experiment, AAT was directly compared to the conventional EEG sleep stages. A total of 15 arousals from 5 subjects were analyzed. These arousals were chosen so that stage REM sleep would be excluded and so that each subject would have one arousal from stage wakefulness, NREM 2 sleep, and NREM 3 sleep. By focusing on NREM sleep, this created a fairer comparison because sleep depth as measured by stage and AAT would both approximate a monotonic function. Using these arousals, two separate statistical analyses were performed with AAT and stage. A conjunction analysis was then performed to compare the significant results. The endpoint of interest was pixels in the correlation matrices and voxels in the brain maps. One color was assigned to pixels/voxels only significant for the analysis with the discrete variable of stage, another color was assigned to pixels/voxels only significant for the analysis with the continuous variable of AAT, and a third color was assigned to overlapping pixels/voxels.

For the network analysis of stage, two linear mixed effects models were created: one with the continuous variable of AAT and the second with a set of dummy-coded variables coding stage as the predictor variables. The latter is automatically performed with MATLAB’s fitlme function by entering the discrete variable into the model specification.

A similar approach was used for the seed-region analysis. The only difference was that stage was analyzed with a repeated-measures analysis of variance. The main effect of AAT was examined with chi-square with two *df*, whereas an *F*-test of the main effect of stage was performed with initial *df*_*effect*_ = 2 and *df*_*error*_ = 8. These were adjusted to 1.8 and 7.2 with the Hyunh-Feldt correction to correct for violations of the assumption of sphericity in repeated-measures data.

Nearly all other parameters were identical to the analyses from Experiment 1. The only other exception was one-tailed tests were used for both AAT and stage because directionality was not important. Comparing the significance of the overall variance explained between AAT and stage was the outcome of interest in this experiment.

### Statistical Conventions

Data are presented as *M ± SD*. All probability thresholds (*α*) are 0.05. To facilitate a better spatial interpretation, some have recommend using *α* = 0.001 as the primary threshold (i.e., before multiple testing correction) in neuroimaging analyses so that clusters of statistically significant voxels do not span multiple neuroanatomical regions (Woo et al., 2014). As an orthogonal and better solution, unthresholded data were prepared to complement thresholded data to minimize the practice of dichotomizing results (Chen et al., 2022; Cohen, 1994; Taylor et al., 2023). For a similar reason, effect sizes are presented to complement null hypothesis testing (Chen et al., 2017; Keppel, 1991).

## Results

### Descriptive Results

The mean age of the subjects was 24.0 (*SD* = 3.5), and 33.3% were male. The amount of excluded data due to motion across the 77 four-min segments was 11.96 ± 22.03 s. Table 1 shows the amount of each sleep stage present in the four-min segments.

### Experiment 1

**Figure 1.**
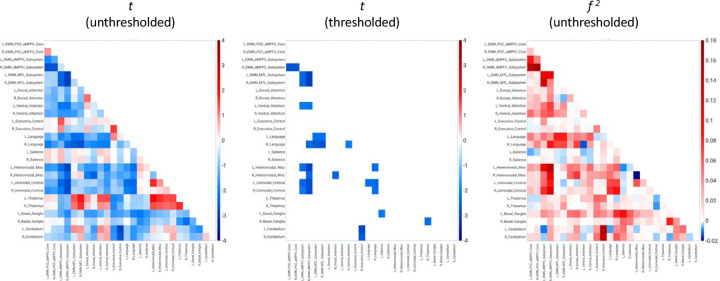
Unthresholded and thresholded linear mixed effects correlations between all auditory arousal thresholds (*n* = 77) and all pairs of network correlations for all subjects (*n* = 12). Thresholded results use only an individual-pixel probability threshold of 0.05; multiple testing correction results are not shown because few if any pixels survived this correction step. *t* = *t*-test. *f* ^2^ = unthresholded Cohen’s *f* ^2^ effect sizes. DMN PCC aMPFC = default-mode network posterior cingulate cortex-anterior medial prefrontal cortex. DMN dMPFC = default-mode network dorsal medial prefrontal cortex. DMN MTL = default-mode network medial temporal lobe.

For the network analysis, Figure 1 displays unthresholded statistical tests, thresholded statistical tests, and effect sizes for the correlation between AAT and the correlation between any two networks. Thresholded results use only an individual-pixel *α* = 0.05; multiple testing correction results are not shown because few if any pixels survived this correction step. It can be seen that the corticocortical correlations were negative. This means that as AAT/sleep depth increased, corticocortical functional correlations decreased.

**Figure 2.**
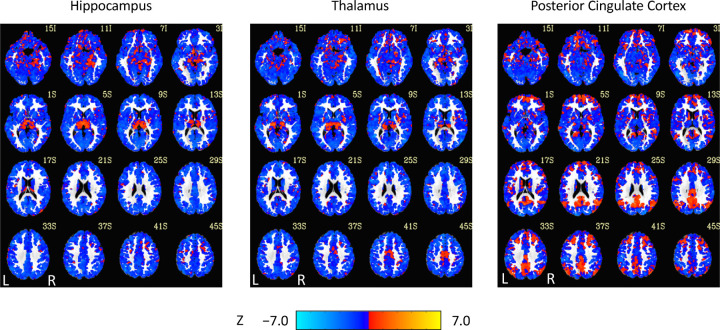
Unthresholded linear mixed effects correlations between auditory arousal thresholds and seed-region correlations for three seed regions with a white matter plus cerebrospinal fluid mask.

**Figure 3.**
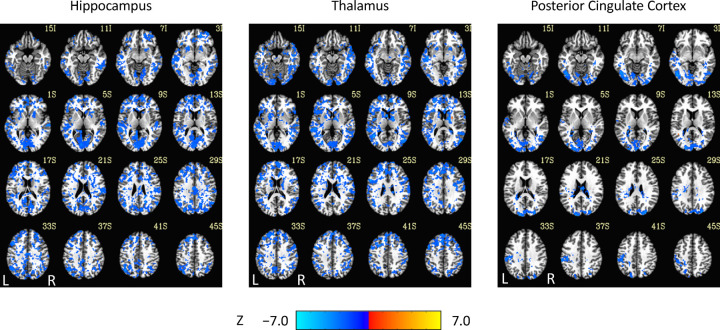
Thresholded linear mixed effects correlations between auditory arousal thresholds and seed-region correlations for three seed regions. A two-sided test, an individual-voxel probability threshold of 0.05, a cluster-size probability threshold of 0.05, and a clustering technique of nearest neighbor with 26 face, edge, and cornerwise neighbors were used. This gave a minimum cluster size of 1,226, 1,195, and 1,203 voxels for the hippocampus, thalamus, and posterior cingulate cortex, respectively. 7

For the results of the seed-region analysis, see Figures 2 and 3. Effect sizes are not shown because most voxels were within *±* 0.0001. The decreases in corticocortical correlations observed at the network level can be observed in the posterior cingulate cortex correlations. For the hippocampus, similar results were found, and this is not surprising given that the hippocampus is often considered part of the default-mode network (Andrews-Hanna et al., 2010). Less expected were the positive correlations between the hippocampal seed region and the thalamus (Figure 2). Although they did not survive statistical thresholding (Figure 3), it is interesting to note them because they are consistent with the unthresholded results in the correlation matrices between the thalamus and the default-mode network medial temporal lobe subsystem. More generally, as AAT/sleep depth increased, thalamic correlations with the neocortex decreased. This was seen in the unthresholded network analysis for the dorsal medial prefrontal cortex subsystem of the default-mode network and the executive control network and in the seed-region analysis widely. This replicates prior work in another dataset demonstrating reduced thalamocortical connectivity during NREM sleep (Picchioni et al., 2014).

**Figure 4.**
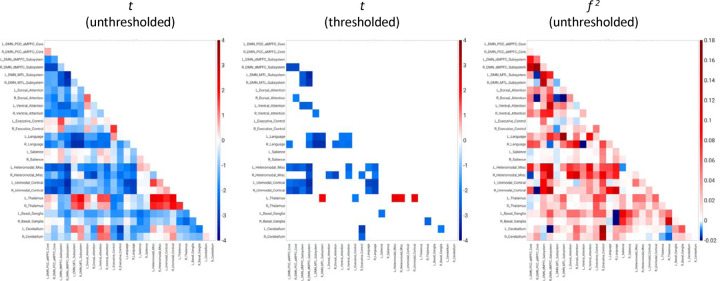
Unthresholded and thresholded linear mixed effects correlations between auditory arousal thresholds excluding stage REM sleep (*n* = 73) and all pairs of network correlations for all subjects (*n* = 12). Thresholded results use only an individual-pixel probability threshold of 0.05; multiple testing correction results are not shown because few if any pixels survived this correction step. *t* = *t*-test. *f* ^2^ = unthresholded Cohen’s *f* ^2^ effect sizes. DMN PCC aMPFC = default-mode network posterior cingulate cortex-anterior medial prefrontal cortex. DMN dMPFC = default-mode network dorsal medial prefrontal cortex. DMN MTL = default-mode network medial temporal lobe.

A concern for these results is the effect of stage REM sleep. One of the primary goals of performing all-night functional MRI sleep studies was to obtain the full spectrum of sleep stages, conventional or undiscovered, during a night of human sleep. The known diversity of brain activity and connectivity within each conventional EEG sleep stage is the reason the current study was performed, and this variance is valuable for searching for undiscovered patterns in sleep. Nevertheless, to compare the current results to prior studies, the majority of which did not have any stage REM sleep, the analysis was repeated while excluding the four arousals from this stage. Figure 4 shows results when excluding these four arousals. Aside from new pixels that passed the threshold mask, including a few positive correlations for the left thalamus, the results were similar.

### Experiment 2

**Figure 5.**
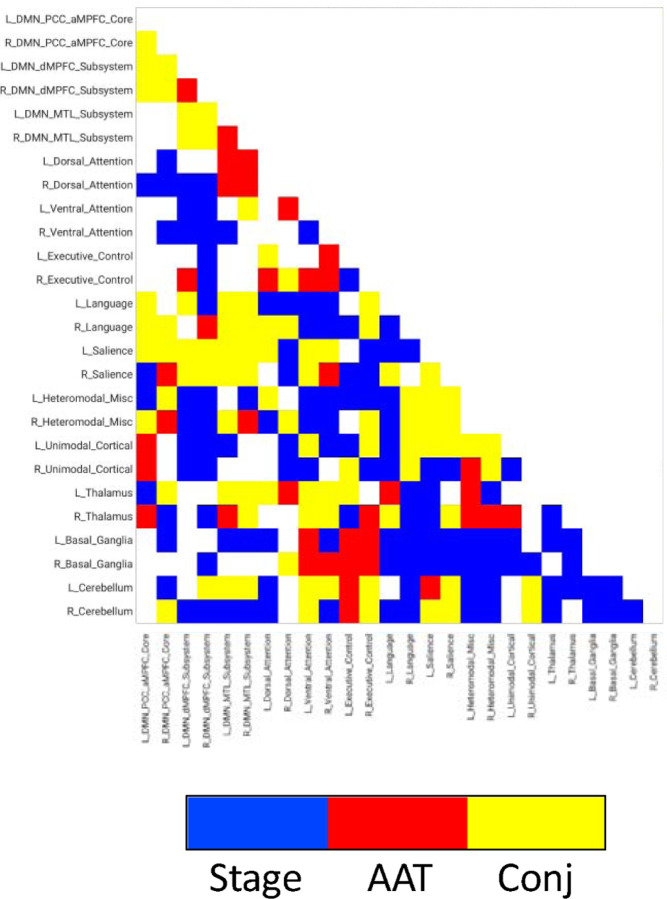
Network results of the conjunction analysis of significant pixels for the discrete variable of stage and the continuous variable of AAT. Stage = stage wakefulness, stage NREM 2 sleep, or stage NREM 3 sleep. AAT = auditory arousal threshold. Conj = conjunction.

**Figure 6.**
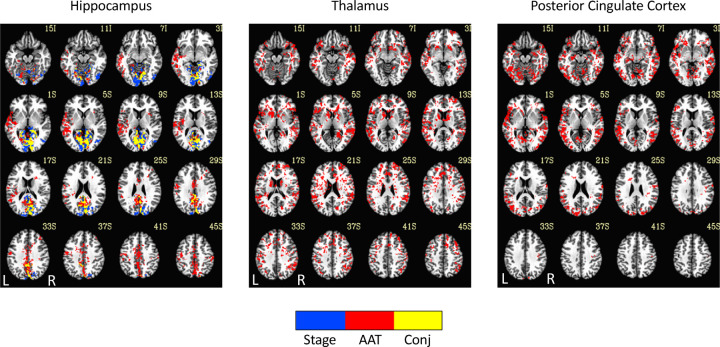
Seed-region results of the conjunction analysis, which compared the overlap between significant voxels for the discrete variable of stage (F_[1.8,7.2]_) using a subsample of the arousals (*n* = 15) and subjects (*n* = 5) and the continuous variable of AAT $\left(\chi_{2}^{2}\right)$ using the same subsample of arousals and subjects. Stage = stage wakefulness, stage NREM 2 sleep, or stage NREM 3 sleep. AAT = auditory arousal threshold. Conj = conjunction.

See Figure 5 and 6 for the results. Numerous pixels/voxels were uniquely significant for AAT compared to those that were only significant for sleep stage or significant for both. This supports the idea that AAT provides unique information above and beyond the conventional EEG sleep stages. This was true for specific pairs in the network analyses and for clusters in the seed-region analysis. These findings indicate that unique patterns of brain connectivity exist within human sleep that are independent from conventional sleep staging.

## Discussion

### Overview

It was hypothesized that, during sleep (defined by high AATs) compared to wakefulness (defined by low AATs), corticocortical functional correlations would decrease. This hypothesis was supported and replicates prior research. Because sleep was defined with arousal thresholds in a way that was independent of the conventional EEG sleep stages, this was a novel approach to studying the neuroanatomical correlates of sleep with high spatial resolution. This work provides initial evidence to justify searching for sleep stages that are more neuroanatomically localized and unrelated to the conventional EEG sleep stages.

Arousals were scheduled randomly. During data collection, the importance of this approach became obvious. EEG data could be manually sleep scored in real-time because real-time MRI artifact corrected EEG data were available (Moehlman et al., 2019). There were several instances of low AATs during what would otherwise be considered EEG-defined stage NREM 3 sleep and high AATs during what would otherwise be considered EEG-defined wakefulness. This variability is known (e.g., Rechtschaffen et al., 1966), so it was not analyzed in the current study, but it is prudent to reiterate it. Moreover, using randomly scheduled arousals to move beyond the conventional EEG sleep stages is novel but not unprecedented. Others have used randomly scheduled arousals, for example, in the context of studying the high-density EEG correlates of dreaming (Siclari et al., 2017) and summarized the importance of this approach by saying investigators need “to move beyond the REM-NREM sleep dichotomy and beyond traditional sleep staging” (Nir & Tononi, 2010, p. 95).

The finding of a decrease in corticocortical correlations with decreases in consciousness (i.e., increases in AAT) replicates numerous other studies using various methods and model organisms, including those that do not rely on blood flow as a proxy for neural activity. Examples include human studies of natural sleep using low-density scalp EEG (De Gennaro et al., 2001), high-density scalp EEG (Titone et al., 2024), intracranial EEG (Bastuji et al., 2021), functional MRI (Horovitz et al., 2009; Picchioni et al., 2013), and simultaneous EEG-transcranial magnetic stimulation (Massimini et al., 2005). These results have been extended to human studies of coma and anesthesia (Noirhomme et al., 2010) and human research on intracerebellar-cortex correlations (Liu et al., in press). This finding is not limited to humans. Decreases in corticocortical correlations have been seen in observational studies of natural sleep using coordinated firing rate fluctuations in excitatory cortical neurons (Olcese et al., 2016), in experimental studies where inhibiting default-mode network nodes caused decreases in corticocortical connectivity and increases in quiet wakefulness (Tu et al., 2021), and in simulated neural networks where NREM sleep depth is modeled by decreasing cholinergic tone (Deco et al., 2014). The current results with AAT are a replication and novel extension because they indicate the same results can be obtained without using the EEG.

It was also important to perform a direct comparison between AAT and sleep stage so that something could be said (neuroscientifically) about the differences in the results. Figures 5 and 6 conveyed details on the functional correlations that would have been missed if one were to perform this analysis with only the conventional sleep stages. For example, the thalamus showed unique relationships with neocortical regions in both the correlation matrix and seed-region results.

### Weaknesses and Strengths of the Current Study

Because functional MRI measures neural activity via blood flow, it is possible that flow could be affected by other factors such as autonomic nervous system tone. This is a limitation for all such studies but is important for the current study because there is substantial variability in autonomic tone throughout sleep (Duyn et al., 2020). This was addressed in the current study by measuring autonomic tone with peripheral vascular volume and respiratory flow rate and removing the variance in the functional MRI signal associated with these variables. These measures must be considered as approximate indicators of peripheral vascular volume and respiratory flow rate. They are roughly proportional to these autonomic processes but are by no means quantitative. It cannot be guaranteed that changes in autonomic tone were effectively removed from the functional MRI signals. However, the results are consistent with the many published studies discussed above. These studies report decreases in corticocortical correlations during sleep with neural measures that do not rely on the neurovascular system, suggesting that the decreases in corticocortical correlations observed with increasing arousal threshold in the current study are neural in origin.

The scanning requirements associated with acquiring MRI data in the relatively short time window (~ seconds) during functional MRI scans cause geometric distortion in the images from inhomogeneities in the static magnetic field. Much of this is controlled with shim fields, but residual distortion can cause unreliable signals near air-tissue boundaries where inhomogeneities are the worst. To mitigate this limitation, spatial smoothing was performed for the seed-region analyses. For the network analyses, voxels were averaged across a large spatial extent for each network. This can be said to take the place of spatial smoothing and provide a similar control for residual distortion. Data collection for future studies will include a phase/field map so that attempts can be made to correct the residual distortion explicitly.

Blood oxygen level dependent functional MRI is well-suited to measure functional correlations because it is a relative measurement and can repeatedly sample brain activity noninvasively at a temporal resolution that, while inferior to that of EEG, is still quite good. These functional correlations are assumed to reflect communication between brain regions, but this cannot be guaranteed because a third region could cause activity in both regions. Further research could attempt to address this limitation with statistical techniques that account for the activity in all brain regions simultaneously.

Not all the arousals in the current study were random. Half were biased towards stage NREM 3 sleep to increase the number of high AATs for these pilot data. This is a limitation because it constrained the variety of sleep states and underlying brain activity/functional correlations patterns. This was the primary gap in the literature that the current study aimed to address. Future studies will use all random arousals.

It cannot be said that the sample size was small in this pilot study. The necessary sample size to detect the effect of interest was not known because an *a priori* statistical power analysis was not performed. The current study was used to calculate the predicted effect sizes for just such a power analysis for a subsequent main study. With the data from the main study, other analyses can be performed, such as including both sleep stage and AAT in the same model to measure the unique variance explained by AAT. This would complement the spatial conjunction analysis reported in the current study.

In the results from the network analyses, few if any pixels survived the multiple testing correct provided by the Network-Based R-Statistics package. This is probably because its correction is overly conservative. It will be important in future studies to use a more-balanced correction.

Common to all studies that use linear correlations, curvilinear relationships between AAT and functional correlations could have been missed. Future studies should include alternative techniques such as polynomial regression, entropy (Watanabe et al., 2014), information-theory measures (Olcese et al., 2016), or distance correlations (Bolt et al., 2024). However, each of these measures have limitations of their own, so they must be considered as complementary to their linear equivalents.

In terms of strengths, with very few exceptions (Picchioni, Ozbay, et al., 2022; Yang et al., 2024), all of which are based on the same data as the current study, analyses performed on all-night functional MRI data do not exist. Moreover, most sleep neuroimaging studies used sleep deprivation, scanned for short durations, and/or began scanning at times that would cause circadian misalignment. These methods and the amounts of sleep obtained with them restrict the generalizability of the results, limiting it to diurnal sleep, a limited number of sleep stages (conventional or otherwise), the recovery sleep that occurs after sleep deprivation, the specific sleep stages that occur at different parts of the night, and/or a single sleep cycle. The current analyses were performed on data without these limitations.

By using arousal thresholds, the current work avoided the circularity associated with studying brain activity/functional correlations associated with the conventional EEG sleep stages. This is an important consideration because undiscovered brain activity/functional correlation patterns may exist during sleep, and only alternative approaches like those employed in the current study could expose such patterns.

### Comparisons with Existing Literature

Investigators have attempted to score the conventional EEG sleep stages with functional MRI data (Tagliazucchi & Laufs, 2014; Tagliazucchi et al., 2012). The method entailed training a machine learning classifier to recognize the functional MRI pattern associated with the NREM EEG sleep stages. The classifier performed well when tested on new data without sleep stage labels and is an essential approach that should be standard in resting-state studies. This method is mostly useful for detecting the conventional EEG sleep stages when simultaneous EEG-functional MRI was not performed. Its ability to say something new about sleep is limited.

Researchers have attempted to localize brain correlates of sleep in a data driven manner with data reduction techniques. Houldin and colleagues (Houldin et al., 2019) performed a spatial independent component analysis of functional MRI data during sleep, and the data were entered into the model regardless of sleep stage. Others have searched for novel spatial brain states during sleep using a Hidden Markov Model (Stevner et al., 2019; Yang et al., 2024). This model characterizes unique brain states based on time series and infers the transitions between those states. Although the investigators used the conventional EEG sleep stages to validate the results, the models were blind to sleep stage when the states and transition probabilities were determined. Like the current study, these approaches are necessary if one hopes to uncover ultradian cycles of novel sleep stages and/or trends of those novel stages across the night.

### Further Research

Data reduction/machine learning techniques can be used in other ways to guide the discovery of novel sleep stages. It may be prudent to enter the conventional EEG sleep stages into the model but examine residual rather than explained variance. Any patterns thus found would be interesting if they were reliably found across the night or across individuals.

The current study was a proof-of-concept. Its analysis focused on the four minutes before the first tone to ensure the functional correlations would be tightly associated with the brain state that led to the arousal threshold. This means only a minority of available data were used. Further research and analyses should capitalize on the full amount of data, for example, to search for novel states in a data driven way across the entire night. In addition to data reduction/machine learning techniques that consider the entirety of the data at once, a sliding window could be used to match a template of a pattern-of-interest across the night. This has been successfully employed during sleep onset (Chang et al., 2016) but could be employed across the full range of sleep states with data like those from the current study.

Another potential avenue for further research comes from the idea of personalized networks. A personalized network is the spatial variability present in a network across subjects. These networks resemble the group average but display differences along their borders, and these differences reliably correlate with differences in cognition between individuals and within individuals across development (e.g., Cui et al., 2020). The canonical resting-state networks found during wakefulness have been spatially correlated with networks from sleep (Houldin et al., 2019). The idea was that networks with low spatial correlations would represent resting-state networks that are unique to sleep. No such networks were found, but low-correlation networks consisting of smaller constituent regions of the canonical networks existed. Perhaps this change in spatial extent is linked to the functions of sleep. For example, such condensed networks could be memory representations that are being refined and optimized for more efficient storage (Tononi & Cirelli, 2014).

Rather than merely searching for neural patterns that are reliably found across the night or across individuals, discoveries could also be made by correlating neural patterns with sleep’s known evolutionarily adaptive cognitive functions such as memory processing (Stickgold & Walker, 2013). This will enable the interpretation of sleep brain activity/functional correlations in a particular region in a less arbitrary way, in comparison to the conventional EEG sleep stages, which were formed without regard to sleep function. The activity/functional correlation patterns that correlate with sleep’s cognitive functions could themselves be considered a new sleep stage, and the cognitive consequences of sleep disturbances in brain diseases/disorders could then be understood by measuring alterations in a functionally defined sleep stage that is connected to its specific symptomology. Stages could then be abandoned, and normative continuous values could be established with an appropriate dataset.

## Conclusion

The current study represents a single step towards a neuroanatomically localized sleep scoring system. If such a system could be developed, then it would not be surprising to discover that additional sleep stages exist given the complexity of brain activity during sleep. Although such stages would need to be unique from wakefulness states to call them a sleep stage, this will expand our understanding of sleep and its functions beyond the constraints imposed by PSG-defined stages.

## Figures and Tables

**Table 1:** Descriptive Statistics from the Conventional Sleep Staging

Stage	*M*	*SD*
W (s)	48.9	83.1
N1 (s)	22.7	44.0
N2 (s)	83.1	86.9
N3 (s)	72.9	94.3
R (s)	12.4	49.5

Note: *n* = 77. W = stage wakefulness; N1 = stage nonrapid eye movement 1 sleep; N2 = stage nonrapid eye movement 2 sleep; N3 = stage nonrapid eye movement 3 sleep; R = stage rapid eye movement sleep.

## Statement of Significance

Conventional sleep stages were established because they correlated with the original behavioral characteristics of sleep, most notably, arousal threshold. With the proliferation of new techniques to measure the brain during sleep, the first experiments might have been to perform correlations with arousal threshold. These experiments have never been performed, either with functional magnetic resonance imaging (MRI) or with any other modern technique. To begin to search for new sleep stages, the amount of communication between brain regions as measured by all-night functional MRI was correlated with arousal threshold. Communication between brain regions decreased as sleep depth, measured behaviorally, increased. This provides initial evidence to justify searching for sleep stages that are unrelated to the conventional sleep stages.

## Abbreviations

AAT: auditory arousal threshold
EEG: electro-encephalography
MRI: magnetic resonance imaging
NREM: non rapid eye movement
PSG: polysomnography
REM: rapid eye movement
